# The Osteogenesis Imperfecta Type V Mutant BRIL/IFITM5 Promotes Transcriptional Activation of MEF2, NFATc, and NR4A in Osteoblasts

**DOI:** 10.3390/ijms23042148

**Published:** 2022-02-15

**Authors:** Vincent Maranda, Marie-Hélène Gaumond, Pierre Moffatt

**Affiliations:** 1 Shriners Hospitals for Children—Canada, Montreal, QC H4A 0A9, Canada; vincent.maranda@mail.mcgill.ca (V.M.); mhgaumond@shriners.mcgill.ca (M.-H.G.); 2 Department of Human Genetics, McGill University, Montreal, QC H3A 0C7, Canada; 3 The Research Institute of the McGill University Health Centre, Montreal, QC H3H 2R9, Canada

**Keywords:** osteoblast, BRIL, IFITM5, osteogenesis imperfecta type V, signaling, NFATC, MEF2, NR4A, luciferase readout

## Abstract

BRIL (bone restricted ifitm-like; also known as IFITM5) is a transmembrane protein expressed in osteoblasts. Although its role in skeletal development and homeostasis is unknown, mutations in BRIL result in rare dominant forms of osteogenesis imperfecta. The pathogenic mechanism has been proposed to be a gain-of or neomorphic function. To understand the function of BRIL and its OI type V mutant (MALEP BRIL) and whether they could activate signaling pathways in osteoblasts, we performed a luciferase reporter assay screen based on the activity of 26 transcription factors. When overexpressed in MC3T3-E1 and MLO-A5 cells, the MALEP BRIL activated the reporters dependent on MEF2, NFATc, and NR4A significantly more. Additional co-transfection experiments with MEF2C and NFATc1 and a number of their modulators (HDAC4, calcineurin, RCAN, FK506) confirmed the additive or synergistic activation of the pathways by MALEP, and suggested a coordinated regulation involving calcineurin. Endogenous levels of *Nr4a* members, as well as *Ptgs2*, were upregulated by MALEP BRIL. Y2H and co-immunoprecipitation indicated that BRIL interacted with CAML, but its contribution as the most upstream stimulator of the Ca^2+^-calcineurin-MEF2/NFATc cascade was not confirmed convincingly. Altogether the data presented provide the first ever readout to monitor for BRIL activity and suggest a potential gain-of-function causative effect for MALEP BRIL in OI type V, leading to perturbed signaling events and gene expression.

## 1. Introduction

Osteogenesis imperfecta (OI) is a rare heritable connective tissue disorder mainly characterized by bone fragility, low bone mass, and short stature. The most severely affected patients suffer from debilitating skeletal deformities and multiple fractures [1,2,3]. Approximately 85% of OI patients have autosomal dominant mutations in *COL1A1* and *COL1A2* [1,2,3]. The only dominant OI types not caused by mutations in *COL1* genes harbor mutations in the interferon induced transmembrane protein 5 gene (*IFITM5* hereafter designated *BRIL* (bone-restricted Ifitm-like)). There have been five genetic variants in *BRIL* reported so far: c.-14C>T (p.M1ext-5) [4,5] in OI type V; c.119C>T (p.S40L) [6,7] and c.119C>G (p.S40W) [8,9] in atypical type VI; and the following two most recent that have not yet been classified (c.143A>G (p.N48S) [10] and c.-9C > A (p.M1ext-3) [11]).

OI type V is estimated to be the second most prevalent form of OI [12], with about 200 cases reported worldwide [4,5,13,14,15]. OI type V is caused by the single recurrent mutation (c.-14C>T) in the 5’ untranslated region of BRIL, leading to the creation of an in-frame ATG with the addition of 5 amino acids (MALEP) at the N-terminus of BRIL [15,16]. The clinical presentation of OI type V resembles OI caused by COL1 mutations with regard to fracture incidence, long-bone deformities, vertebral compression fractures and scoliosis [17]. But it presents a most distinguishing clinical feature that is the formation of hyperplastic callus, which can occur spontaneously or after fractures in long bones [18,19]. Callus formation coincides with episodes of severe inflammation and increased serum alkaline phosphatase (ALP) activity, suggesting a high rate of osteoblastic differentiation and bone formation [17]. Other specific radiographic findings include new periosteal bone formation along the interosseous membrane of the forearm. As visualized at the histological level under polarized light, the bone matrix has a mesh-type lamellation pattern [15,20]. The bone histological features of OI-V indicate disturbances in osteoblast function on both endosteal and periosteal bone surfaces, with an hypermineralized bone matrix and a high osteocyte density, possibly suggesting exuberant but abnormal bone formation [20].

BRIL is part of an evolutionary conserved family of so-called small IFITM proteins [21,22], but has evolved from a distinct earlier ancestor, implying divergence of function [23]. BRIL is a 132 a.a. type II transmembrane protein that localizes mostly to the plasma membrane and Golgi [24]. It is post-translationally S-acylated [24,25] through covalent attachment of 16-carbon chain palmitoyl-coA on 3 cytoplasmic cysteines. Expression of BRIL is mostly restricted to osteoblasts and is highly expressed in developing intramembranous and endochondral bones, but not in chondrocytes [16]. Recent single cell RNAseq analyses have systematically reported *Bril* as being an exquisite marker of osteoblasts [26,27,28,29], and potentially also part of a gene signature of osteocytes [30].

Though there is convincing genetic evidence for the importance of the pathogenic role(s) of BRIL in humans, its physiological function is still unknown. Even though gain- and loss-of-function of BRIL in cellulo have suggested a role in osteoblast mineralization [16], this was not corroborated in vivo as overexpression and knockout mouse models did not show any developmental or post-natal mineralization defects in their skeleton [31,32,33]. It is unknown whether the absence of BRIL in humans is detrimental or not. Mice carrying the -14C>T mutation, generated either through transgenesis [34] or CRISPR-CAS9 editing [35,36], displayed severe skeletal malformation and did not survive past birth. Both the WT and mutant MALEP forms of BRIL were produced in osteoblasts and their differentiation was compromised and blunted very early (E14.5) during embryogenesis [35]. In line with the inflammatory episodes observed in OI type V patients [18,37,38], the MALEP BRIL mice had a significant upregulation of the prostaglandin synthase enzyme COX-2 (encoded by *Ptgs2*), indicating that this may partly be associated with pathophysiology [35]. The absence of skeletal defects in the *Bril* knockout mouse models, combined with the equal expression of the mutant allele in OI type V patients [24,39], provided strong evidence that OI type V is not caused by haploinsufficiency, but by a gain of (detrimental) function. However, the cellular and molecular mechanism(s) by which the MALEP BRIL mutant is causing OI type V remains largely elusive.

The goal of the current study was to broadly investigate the potential biological function of wild type (WT) BRIL, and to examine the unique or common activity driven by the OI type V pathogenic variant MALEP BRIL. We sought primarily to investigate whether the BRIL forms could elicit intracellular signaling pathways in osteoblasts. A non-biased screen was conducted for the ability of WT and MALEP BRIL to induce the activation of luciferase (Luc) reporters transcriptionally driven by multimerized copies of DNA binding element for 26 different transcription factors. The screen was performed by co-transfection assays in the pre-osteoblastic MC3T3-E1 and pre-osteocytic MLO-A5. Further analyses were conducted to examine in more detail the signaling pathways and intermediate players involved with activation of 3 reporters: MEF2, NAFTc, and NR4A. Elucidating these new functions of BRIL and mutant MALEP are important to better understand how they might impact osteoblast activity, and consequently lead to OI. Shedding light on the nature of these mechanisms could potentially lead to ways to suppress them and pave the way to new therapeutic avenues for OI type V.

## 2. Results

### 2.1. BRIL Induces Pathways Leading to Activation of the MEF2-, NFATc-, and NR4A-Luc Reporters

**Figure 1:**
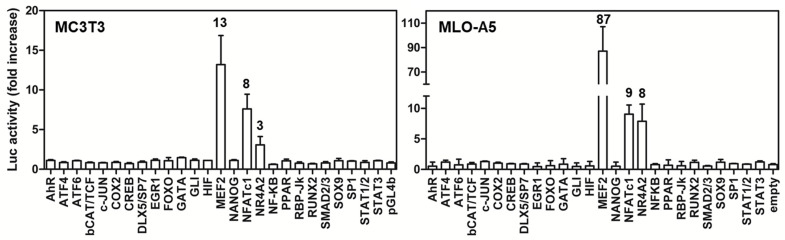
Screen of luciferase reporter activity for WT BRIL in MC3T3-E1 and MLO-A5. Cells were co-transfected with 100 ng of CMV-based expression plasmids (mouse BRIL or GFP), and 100 ng of each of the indicated luciferase (Luc)-reporter constructs. Twenty-four hours later, the Luc activity was measured, and the activity elicited by BRIL was normalized to that of the baseline activity obtained with a GFP expression plasmid. Fold-increases above GFP are indicated above bars. Data represent the average ± SD (*n* = 3).

The physiological function of BRIL in osteoblast biology is still unclear. To explore whether BRIL can stimulate an intracellular signaling response, we conducted a screen for its ability to induce the activation of luciferase (Luc) reporters. Each of the 26-reporter plasmids tested has multimerized copies of a DNA binding element for a given transcription factor (see Supplemental Table S1), cloned upstream of a minimal promoter that drives expression of firefly Luc (Figure 1). Increased reporter Luc activity therefore serves as a surrogate readout to identify a signaling pathway stimulation culminating in a transcriptional response. The reporters were selected to cover a range as wide as possible to reflect of different pathways active in bone cells. The screen was performed in the pre-osteoblastic MC3T3-E1 [40] and late-osteoblastic MLO-A5 [41]. As compared to a plasmid expressing GFP, mouse BRIL induced a substantial upregulation of the MEF2, NFATc, and NR4A2 reporter constructs, in both lines, albeit less for NR4A2 in the MC3T3-E1. All other reporters tested remained at baseline. For NFATc and NR4A2, the magnitude of responses obtained in both lines were comparable, whereas that obtained for MEF2 was much greater in the MLO-A5 (87-fold) than in the MC3T3-E1 (13-fold).

### 2.2. The Mouse and Human Wild Type (WT) and Mutant (MALEP) BRIL Activate MEF2- and NFATC-Luc Reporters in MC3T3-E1

**Figure 2:**
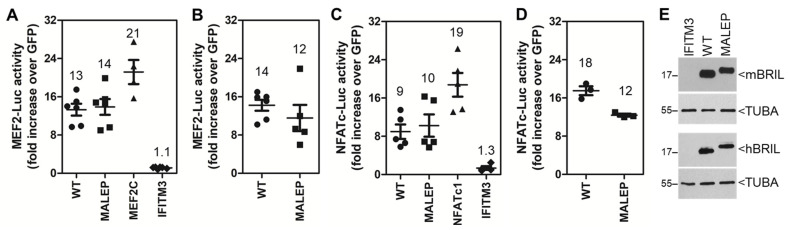
Comparison of the activity of the mouse and human BRIL on MEF2-Luc and NFATc-Luc in MC3T3-E1. The indicated expression plasmids were co-transfected (50 ng each) with 100 ng of the MEF2-Luc (**A**,**B**) and NFAT-Luc reporters (**C**,**D**) in MC3T3-E1 cells and Luc activity was measured 24 h later. (**A**,**C**) The mouse wild type (WT) and MALEP BRIL forms were compared to the human (**B**,**D**). All results were normalized to a GFP-expressing plasmid. Data represent average ± SEM (*n* = 3–6), as illustrated by the individual datapoints for each set of transfections. (**E**) Western blot detection of mouse and human WT and MALEP BRIL. Equal loading of samples on blots was confirmed by alpha tubulin (TUBA) hybridization.

Because the expression of the mutant MALEP BRIL in OI type V has been proposed as a gain-of-function, we next compared the ability of the wild type (WT) BRIL and its mutant MALEP form at inducing the MEF2-Luc and NFATc-Luc reporters in MC3T3-E1 (Figure 2). These 2 reporters were selected for a more thorough subsequent analysis in the MC3T3-E1, as they showed strongest responses in the Luc assay. The involvement of NR4A was also investigated in the context of the MLO-A5 cells as described further below. The mouse and human BRIL were tested to ascertain conservation of function between the 2 species. Both the mouse (Figure 2A,C) and human (Figure 2B,D) BRIL (WT and MALEP) were equally effective at inducing both reporters, albeit a little less for the human MALEP. The extent of activation by WT and MALEP BRIL was considerable (≥10-fold), as compared to the response elicited by expression of the MEF2C and NFATc1 transcription factors on their own reporters (21- and 19-fold, respectively). Overexpression of IFITM3 was tested as another IFITM-family member, but did not cause any activation of the reporters over the GFP-baseline (Figure 2A,C). Western blotting confirmed that there was equal expression of mouse and human WT and MALEP BRIL (Figure 2E). Because the mouse and human BRIL gave similar effects on the Luc-reporters, only the mouse BRIL species were used in all subsequent experiments.

### 2.3. Overexpression of BRIL Does Not Lead to Increased Transcription of the Mef and NFATc Genes

To verify whether the observed MEF2-Luc and NFATC-Luc reporter activation by BRIL was not caused by a direct increased transcriptional response, the gene expression for the 4 *Mef2* (a, b, c, d) and 3 *Nfatc* (1, 2, 3) genes was analyzed. Real-time qPCR analysis indicated that forced expression of WT or MALEP BRIL in MC3T3-E1 or MLO-A5 did not cause any statistically significant changes in any of the genes monitored (Supplemental Figure S1). The results also revealed which of the possible members endogenously expressed could have contributed to the observed activation in the Luc-reporter activity. In both cell lines the levels of expression for *Mef2* and *Nfatc* were very similar. *Mef2a* was the most highly expressed, followed by moderate levels of *Mef2d* and *Mef2c*, and *Mef2b* was absent in MC3T3-E1 and very low in MLO-A5. Similarly, levels of *Nfatc1* and *Nfatc3* were highest in both lines, but *Nfatc2* was 20- to 100-fold less.

### 2.4. The Ability of WT and MALEP BRIL to Induce MEF2 and NFATc Is Repressed by HDAC4 and FK506

**Figure 3:**
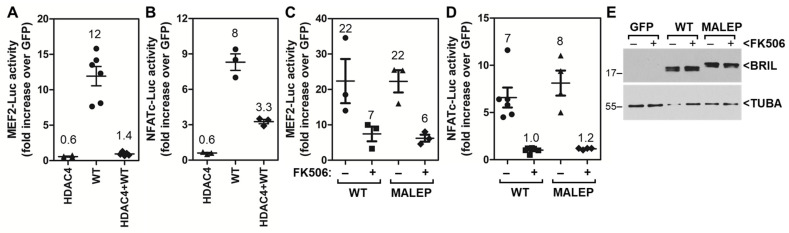
Effects of HDAC4 and FK506 treatment on BRIL-inducing activity in MC3T3-E1. The indicated expression plasmids were co-transfected with the MEF2-Luc (**A**,**C**) and NFAT-Luc reporters (**B**,**D**). The transfection mixes contained 100 ng of Luc-reporters, 70 ng of WT BRIL, and 30 ng of HDAC4 (**A**,**B**). Cells transfected with WT or MALEP BRIL were treated with 10 nM FK506 (+), or DMSO vehicle (-), 3 h post-transfection (**C**,**D**). Luc activity was measured at 24 h and normalized to a GFP-expressing plasmid. Data represent average ± SEM (*n* = 3–6). The average fold increases are indicated above each dataset. (**E**) Western blot detection of mouse WT and MALEP BRIL. Equal loading of samples on blots was confirmed by alpha tubulin (TUBA) hybridization.

The MEF2 and NFATc transcriptional activities are known to be modulated by HDAC4 and calcineurin A (CnA) (also known as protein phosphatase type 2B), respectively. While HDAC4 represses MEF2 by direct protein-protein interaction, CnA is a phosphatase leading to dephosphorylation and activation of NFATc. Considering BRIL had no direct effect on *Mef2* and *Nfatc* gene expression, the next series of experiments were conducted to validate that the stimulatory activity of BRIL on both pathways could be modulated by HDAC4 and CnA. It was also explored whether HDAC4 and CnA were selective for their respective pathway (MEF2 or NFATc) or if there was a possible crosstalk between the two. The MEF2-Luc and NFATc-Luc reporters were significantly reduced below baseline (0.6--fold) by expression of HDAC4 alone in MC3T3-E1 cells (Figure 3A,B). Similarly, HDAC4 totally blocked BRIL-inducing activity to baseline levels (1.4-fold) on MEF2-Luc (Figure 3A), but partially only (~60% decrease) for NFATc-Luc (Figure 3B). These data corroborated that BRIL promoted the activity of endogenous members of MEF2 and NFATc, but also suggested some crosstalk between them.

The requirement for CnA in the activation of the reporters by BRIL was further tested by treating cells with the inhibitor FK506. For this specific context, cells were treated with FK506 (10 nM) or the DMSO vehicle, starting 3 h after transfection until collection at 24 h. FK506 treatment significantly reduced the MEF2-Luc and NFATc-Luc reporters (Figure 3C,D). The reduction was partial for MEF2-Luc (Figure 3C) (~70% decrease), but complete for NFATc-Luc (Figure 3D). The effect of FK506 was equally effective on WT and MALEP BRIL, but did not affect the protein levels of BRIL (Figure 3E).

**Figure 4:**
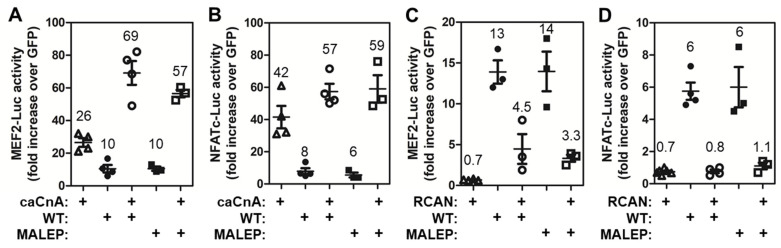
Effects of caCnA and RCAN on BRIL-inducing activity in MC3T3-E1. Wild type (WT) and MALEP BRIL were co-transfected in the presence or absence of a constitutively active form of calcineurin (caCnA) (**A**,**B**) or regulator of calcineurin (RCAN) (**C**,**D**). Each transfection was carried out with a total of 200 ng of plasmid DNA: 50 ng of each effector and 100 ng of reporters. The MEF2-Luc (**A**,**C**) and NFATc-Luc (**B**,**D**) reporter activities were measured after 24 h and normalized to a GFP-expressing plasmid. Data represent average ± SEM (*n* = 3–4). The average fold increases are indicated above each dataset.

To further confirm the identity of the pathway leading to activation of the MEF2-Luc and NFATC-Luc by BRIL, co-transfections were performed with a constitutively active form of CnA (caCnA), or with its negative regulator RCAN. The caCnA is truncated, missing the C-terminal domain for calmodulin interaction and autoregulatory inhibitory region. The RCAN negative feedback regulator binds to CnA and directly inhibits its activity. caCnA and RCAN were tested with WT and MALEP BRIL on both the MEF2-Luc and NFATc-Luc. In MC3T3-E1, caCnA alone significantly upregulated the activity of both MEF2-Luc and NFATc-Luc (Figure 4A,B). When co-transfected with WT or MALEP BRIL, the Luc activity readouts were greater than the sum of caCnA and BRIL activities individually (Figure 4A,B), indicating a synergistic action. On the other hand, RCAN alone slightly (0.7-fold) but consistently brought the reporters activity below baseline (GFP control) (Figure 4C,D). When co-transfected with either BRIL or MALEP BRIL, RCAN reduced the MEF2-Luc by ≥65% (Figure 4C), but completely abrogated their effects on the NFATC-Luc reporter (Figure 4D). In either case, with caCnA or RCAN, there was no significant differences observed between WT and MALEP BRIL forms. Altogether these results suggest that CnA acts downstream of BRIL in a common pathway to stimulate MEF2 and NFATc.

### 2.5. Time-Course and Dose-Response for WT and MALEP BRIL Activity in MC3T3-E1

**Figure 5:**
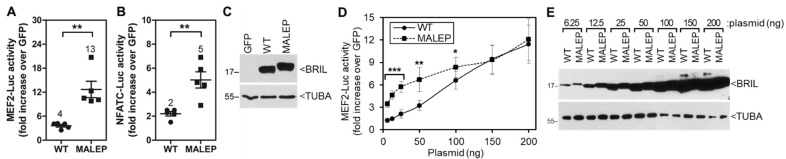
Effects of WT and MALEP BRIL in MC3T3-E1 under sub-saturating conditions. Wild type (WT) and MALEP BRIL were co-transfected with the MEF2-Luc and NFATc-Luc reporters and activities measured after 9 h (**A**,**B**) or 24 h (**D**). (**A**,**B**) Transfections were carried out with 100 ng of plasmid DNA effectors (WT or MALEP BRIL, or GFP) and 100 ng of reporters. Data represent average ± SEM (*n* = 5). The average fold increases are indicated above each dataset. (**D**) Transfections were carried out with increasing amounts of plasmid DNA effectors (BRIL or MALEP) and 100 ng of reporters. All transfection mixes were completed to a total of 200 ng with a GFP expression plasmid. The reporter activities were measured after 24 h and normalized to the GFP-expressing plasmid alone. Data represents average ± SEM (*n* = 4). (**C**,**E**) Western blot detection of mouse WT and MALEP BRIL. Equal loading of samples on blots was confirmed by alpha tubulin (TUBA) hybridization. *** *p* < 0.001; ** *p* < 0.01; * *p* < 0.05.

The gain-of-function of the MALEP BRIL form would have been predicted to produce an increased activity over the WT BRIL. All of the preceding experiments, which gave essentially same readouts for both WT and MALEP, were conducted at steady-state (24 h) with fixed amounts of input plasmids. To better measure potential differences between WT and MALEP BRIL that might have escaped detection under these conditions, the transfection and measurement of the MEF2-Luc and NFATc-Luc reporter assays were refined in 2 different ways. First, the Luc measurements performed at 9 h post-transfection, to capture the earliest possible effects, revealed that MALEP was significantly more active than WT BRIL on the MEF2-Luc (3.2-times more) and NFATc-Luc (2.5-times more) reporters (Figure 5A,B). Western blotting for BRIL protein expression at 9 h indicated identical levels (Figure 5C). Second, a dose-response experiment was performed by increasing the amount of plasmid transfected and measuring the MEF2-Luc activity at 24 h (Figure 5D,E). The readout was significantly greater for MALEP as compared to WT BRIL for plasmid amounts ranging from 6.25 ng to 100 ng (Figure 5D). Beyond 100 ng, the activities for MALEP and WT BRIL were identical, suggesting that the signal had reached saturation. Western blotting also indicated comparable levels for both proteins at all the different amounts of plasmid transfected (Figure 5E). The results obtained under sub-saturating conditions substantiate the concept that MALEP BRIL has a greater potential to activate the pathways and influence the cell activity.

### 2.6. Screening of the Activity of BRIL on MEF2-Luc and NFATc-Luc in Various Cell Lines

**Figure 6:**
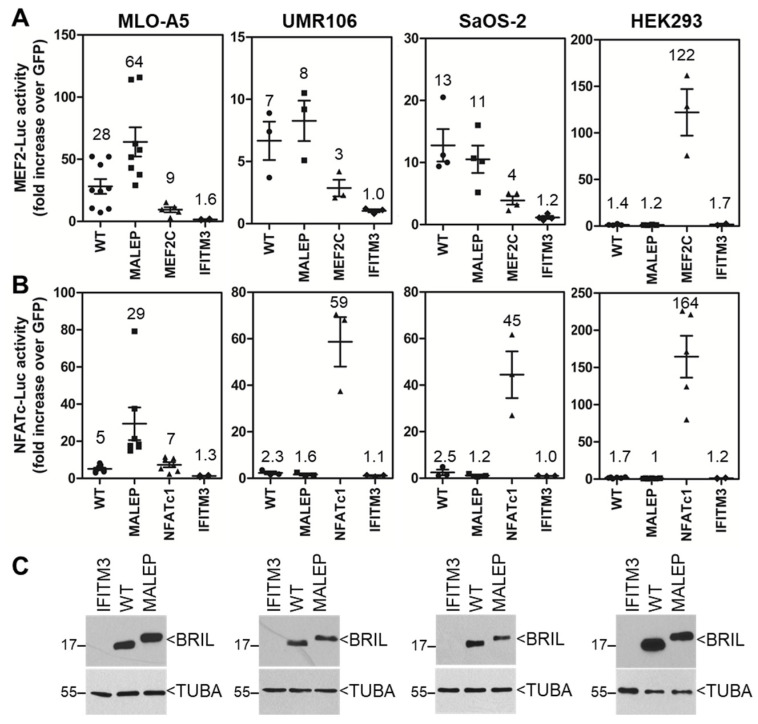
Comparison of the activity of mouse BRIL on MEF2-Luc and NFATc-Luc in various cell lines. The indicated expression plasmids (100 ng) were co-transfected with 100 ng of the MEF2-Luc (**A**) and NFATc-Luc reporters (**B**) in the cell lines indicated above the graphs. Luc activity was measured 24 h later and normalized to a GFP-expressing plasmid. Data represent average ± SEM (*n* = 3–8). The average fold increases are indicated above data points each representing one experiment performed in duplicate wells. (**C**) Western blot detection of mouse and WT and MALEP BRIL are presented below each matching graph for each line. Equal loading of samples on blots was confirmed by alpha tubulin (TUBA) hybridization.

The effect of the WT and MALEP BRIL was next tested in various cell lines, aiming at possibly finding a cellular-context that would be more permissive to find differences between the 2 BRIL forms at steady state. In addition to the pre-osteocytic MLO-A5 cells, for which we had already observed activity for the mouse WT BRIL (Figure 1), 2 osteosarcoma lines (rat UMR106 and human SaOS-2) and 1 embryonic kidney epithelial line (human HEK293) were tested (Figure 6). The WT and MALEP BRIL were active on the MEF2-Luc (Figure 6A) reporter in MLO-A5, UMR106, and SaOS-2, while they were inactive in the HEK293. In the MLO-A5, UMR106, and SaOS-2 cells, WT and MALEP BRIL gave more robust induction folds on the MEF2-Luc than the MEF2C positive control (Figure 6A). As for the NFATc-Luc reporter, it was only significantly activated in the MLO-A5 (Figure 6B). As was observed previously in the MC3T3-E1 cells (Figure 2), the plasmid expressing the mouse IFITM3 did not elicit any response in any of the cells examined. Interestingly, only in the MLO-A5, the MALEP BRIL caused significantly more activation on both reporters than the WT BRIL: 2.3-fold and 5.8-fold more on the MEF2-Luc and NFATc-Luc, respectively (Figure 6A,B). In all cell lines, there was equal production of WT and MALEP BRIL proteins (Figure 6C). Thus, the cellular context (osteoblast) appears to be a determinant of the activity of BRIL, and that the MLO-A5 may be more amenable at steady-state to determine a potential mechanism. It does not preclude, however, that other lines may have also displayed a similar effect of the MALEP under optimized conditions as was done for the MC3T3-E1 cells above.

### 2.7. MALEP BRIL Selectively Induces Gene Expression of Nr4a Members and Ptgs2 in MLO-A5 Cells

**Figure 7:**
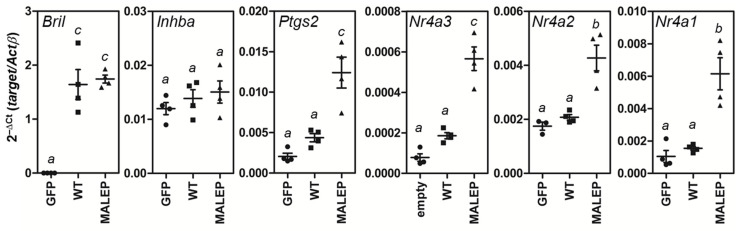
Gene expression monitoring by qPCR in transfected MLO-A5 cells. Total RNA was collected 24 h after transfection with expression plasmids encoding GFP, WT and MALEP BRIL. RT-qPCR was performed to analyze the various targets indicated within each graph. Expression levels are expressed as 2^−^^Δ^^Ct^ as normalized to β-actin (*Actβ*) as indicated on the left panel y-axis. Data represent average ± SEM (*n* = 4). The different letters indicate a statistically significant difference between groups with the following *p* values: a > 0.05; b ≤ 0.01; c ≤ 0.001.

The forced expression of WT and MALEP BRIL in the MLO-A5 also did not significantly cause changes in the endogenous levels for *Mef2* (a, b, c, and d), *NFATc* (1, 2, 3, 4) (Supplemental Figure S2A). Considering the repressive potential of HDAC, overexpressed WT or MALEP BRIL did not cause any significant decrease in expression of 7 *Hdac* genes in the MLO-A5 (Supplemental Figure S2B). Because of the possibly more favorable cellular context of the MLO-A5, the array of gene expression monitoring was expanded to include the 4 genes (*Msmp*, *Inhba*, *Ptgs2* and *Nr4a3*) that we previously demonstrated as being upregulated in the bones of the MALEP BRIL knock-in mouse model [35]. *Bril* overexpression was first validated and showed equal abundance after transfection of WT and MALEP forms (Figure 7). The expression of *Msmp* was undetectable (not shown), whereas *Inhba* was unaffected by BRIL. However, *Ptgs2* and *Nr4a3* were both significantly upregulated when MALEP BRIL was transfected as compared to WT BRIL. Two other members of the *Nr4a* gene family, *Nr4a1* and *Nr4a2*, were equally upregulated by MALEP BRIL. These results indicate that MALEP BRIL expression not only causes activation of ectopic Luc reporters, but also contributes to modulating endogenous gene targets.

### 2.8. Differences in the Activity of WT and MALEP BRIL to Regulate MEF2, NFATc, and NR4A Reporters

**Figure 8:**
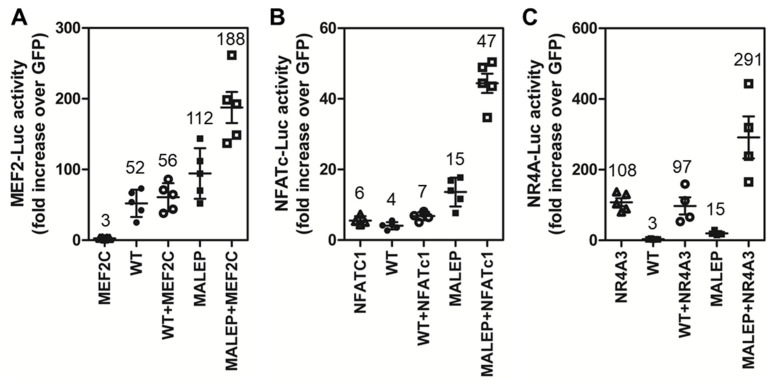
Comparison of the effects of WT and MALEP BRIL in conjunction with MEF2C, NFATC1, and NR4A3 in MLO-A5. The indicated expression plasmids were co-transfected with the MEF2-Luc (**A**), NFAT-Luc (**B**), and NR4A3 (**C**) reporters. Each transfection was carried out with a total of 200 ng of plasmid DNA: 50 ng of each effector (or empty plasmid for single transfection), and 100 ng of reporters. The Luc reporters were measured after 24 h and normalized to the GFP-expressing plasmid. Data represent average ± SEM (*n* = 4–5). The average fold increases are indicated above data points, each representing one experiment performed in duplicate wells.

To further examine the response differences between WT and MALEP BRIL in MLO-A5 cells, co-transfection was performed with or without the MEF2C, NFATc1, and the NR4A3 transcriptional activators (Figure 8). Comparison of the Luc reporter activity was conducted with both BRIL forms (WT vs. MALEP) in the absence or presence of the respective transcription factors (MEF2C, NFATc1, NR4A3). On all 3 reporters, the WT BRIL gave at best additive effects when combined with the respective direct activators MEF2C (Figure 8A; 3 + 52 vs. 56), NFATc1 (Figure 8B; 6 + 4 vs. 7), and NR4A3 (Figure 8C; 108 + 3 vs. 97). The MALEP BRIL, however, gave synergistic effects on the MEF2-Luc (Figure 8A; 3 + 112 vs. 188), NFATc-Luc (Figure 8B; 6 + 15 vs. 47), and NR4A3 (Figure 8C; 15 + 97 vs. 291) pointing to its gain-of-function. These data also suggest that activation of the NR4A-Luc reporter could be due to the observed increased in the *Nr4a* genes expression.

### 2.9. Interaction of BRIL with CAML by Yeast 2-Hybrid and Validation by Co-Immunoprecipitation

The data gathered so far indicate that WT and MALEP BRIL can elicit an intracellular cascade leading to activation of the transcription factors MEF2, NFATc, and NR4A3. In an effort to pinpoint the direct connection between BRIL and an immediate molecule(s) that could relay the signal to the downstream transcriptional effectors, and which may interact with MALEP preferentially, a differential yeast 2-hybrid (Y2H) screen was performed. To make this possible given the type II topological transmembrane configuration of BRIL, only the intracellular cytoplasmic segments of human WT (a.a. 1-86) and MALEP (a.a. 1-91) BRIL were fused to GAL4-DBD as the bait, so that their N-terminus would remain unmasked, as for the natural proteins. A high throughput DNA sequencing approach was taken for the identification of positive hits. A relatively high number of confidence hits were discovered for WT (56) and MALEP (68). Their identity and distribution within 3 pre-defined confidence tiers are listed in Supplemental Table S5. The identity of the high confidence interaction results for WT BRIL were EXOC5, MED21, SCG3, UBQLN1, UBQLN2, and for MALEP BRIL the hits were CAML, MED21, SCG3, UBQLN1, UBQLN2, thus showing a significant overlap. There was no substantial enrichment of preys being differentially captured by MALEP BRIL.

**Figure 9:**
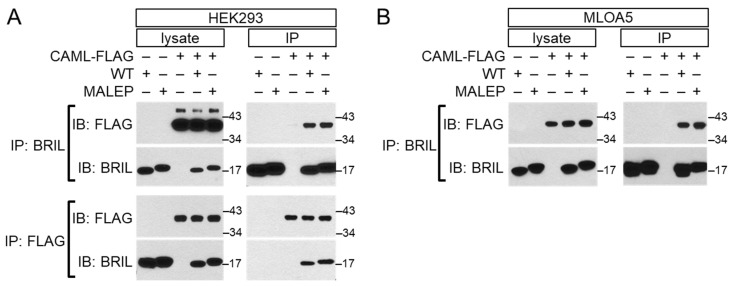
Co-immunoprecipitation of WT or MALEP BRIL with CAML in HEK293 and MLO-A5 cells. Plasmids expressing the C-terminally FLAG-tagged CAML (CAML-FLAG), together with WT or MALEP BRIL, were co-transfected in HEK293 (**A**) andMLO-A5 (**B**) cells. After 24 h, cell lysates were prepared and immunoprecipitated with the anti-BRIL or anti-FLAG antibodies. Both the total lysates (lysate) or immunoprecipitated proteins (IP) were analyzed by Western blotting (IB) with the indicated antibodies. Membranes were hybridized with the first antibody, stripped, and re-incubated with the second antibody. Representative experiments of at least 3 are shown. Molecular mass markers are indicated at right.

Based on different in silico criteria such as expression in bone cells, sub-cellular localization, and putative functional annotation, three of the highest ranking (UBQLN1, CAML, EXOC5) hits were tested for validation by co-expression in HEK293 cells and co-immunoprecipitation (co-IP). Only CAML (calcium-modulating cyclophilin ligand) was detected reproducibly when the pull down was performed with the anti-BRIL antibody (Figure 9A-top). Reciprocally, BRIL was pull-down when an anti-FLAG antibody was used for the pull down (Figure 9A-bottom), and WT and MALEP BRIL similarly interacted with CAML (Figure 9A). Identical results were obtained when the BRIL co-IP was performed in MLO-A5 cells (Figure 9B).

### 2.10. CAML Differentially Regulates the Activity of MEF2 and NFATc

**Figure 10:**
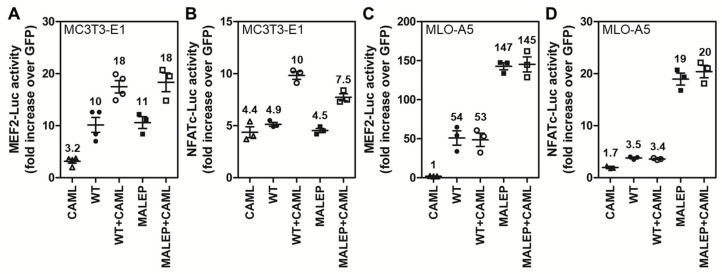
Effect of the combination of CAML with WT and MALEP BRIL on the MEF2-Luc and NFATC-Luc reporters. The indicated expression plasmids were co-transfected with the MEF2-Luc (**A**,**C**) and NFATc-Luc reporters (**B**,**D**) in MC3T3-E1 (**A**,**B**) and MLO-A5 cells (**C**,**D**). Each transfection was carried out with a total of 200 ng of plasmid DNA: 50 ng of each effector (or empty plasmid for single transfection), and 100 ng of reporters. The MEF2-Luc and NFATc-Luc reporter activities were measured after 24 h and normalized to the GFP-expressing plasmid. Data represent average ± SEM (*n* = 3–4). The average fold increases are indicated above data points, each representing one experiment performed in duplicate wells.

CAML was first identified as a molecule activated downstream of CnA and as an activator of the NFATc1 pathway. The possibility that the interaction of WT and/or MALEP BRIL with CAML could mediate the effect on NFATc was next tested with the NFATc-Luc reporter (Figure 10). In the MC3T3-E1, CAML alone had positive effects on the MEF2-Luc and NFATc-Luc reporters, but the combined effects with WT or MALEP BRIL were only additive (Figure 10A,B). In the MLO-A5, CAML alone did not induce a significant activation of the 2 reporters, and had no additional effects when combined with BRIL (Figure 10C,D). In the MLO-A5, as observed above, the effects of MALEP BRIL were significantly more robust than those of the WT (Figure 10C,D). The data would suggest that the interaction of BRIL with CAML, under the current conditions, may not be the main driver of the transcriptional responses.

## 3. Discussion

BRIL is an osteoblast-specific transmembrane protein for which the function in skeletal biology is still unknown. However, BRIL pathologic importance has been exemplified by a distinct set of mutations in dominant OI types. The most frequent by far is the c.-14C>T causing OI type V, which is believed to either result from a gain-of-function or a neomorph. The mechanisms leading to the dominant nature of the encoded MALEP BRIL have remained largely unexplored. In the current study we identified that specific intracellular pathways are activated by the WT and mutant MALEP BRIL. Using a Luc-based screening approach, we found that WT BRIL led to activation of 3 reporters regulated by family members of MEF2, NFATc, and NR4A transcription factors. The MALEP BRIL was found to be chiefly more active at inducing the 3 reporters than WT BRIL, and that appeared to be dependent on the cellular context, under optimal conditions MC3T3-E1, and constitutive in the MLO-A5. A series of co-expression experiments with known modulators of MEF2 (HDAC4) and NFATC (CnA), or treatment with an antagonist of CnA (FK506) revealed some crosstalk between the 2 pathways, and suggested that CnA could be one of the most upstream mediators of this cascade. The direct interaction of BRIL with CAML could represent a potential mechanism involving the modulation of intracellular calcium stores. These results would favor a gain-of-function, as opposed to a potential neomorphic cause, for MALEP BRIL.

Although highly variable, the clinical manifestations in OI type V patients are associated with low bone density, fractures, spinal deformities, radial head dislocation, hyperplastic callus formation, and paradoxical expanded periosteal bone formation that can be accompanied by interosseous membrane calcification [4,5,13,14,15,17]. Although very well described from the clinical perspective, the onset and progression of those events in OI type V are much less well understood at the molecular level. Only a few studies have looked at histological appearance and matrix composition at the tissue level. Histological analyses of hyperplastic callus have revealed very heterogeneous tissue, containing edematous areas with occasional atrophic muscle fibers, hypercellular woven bone, fibrochondroid and chondroid tissue [42,43,44,45,46,47], possibly alluding to perturbed events in mesenchymal stem cells differentiation. The periosteal bone expansion is not always linked to hyperplastic callus, indicating that more subtle intramembranous periosteal events may be involved [15]. The enhanced signaling capability of MALEP BRIL described herein could be compatible with such changes in osteoblastic differentiation and function, caused ultimately by perturbed gene expression relayed by the activated MEF2, NFATc, and/or NR4A family of transcription factors.

The mechanism(s) by which the WT and MALEP BRIL activated the signaling cascades could be caused by overlapping mechanisms. Using co-immunoprecipitation experiments we have not been able to detect any direct interaction between BRIL forms and MEF2C and NFATc1 (data not shown). *Mef2* and *Nfatc* gene expression were also not altered by BRIL. Both transcription factors, however, rely on their phosphorylation status for their entry and subsequent activity in the nucleus, which favors the involvement of CnA as a common mechanism acting just downstream of BRIL for the activation of MEF2 and NFATc. This was supported by the results showing the synergistic effects of BRIL and CnA, and the repressive effects of RCAN1, HDAC4, and the immunosuppressive drug FK506. Previous studies have provided evidence that Ca^2+^-calcineurin is a common player leading to activation of MEF2 and NFATc [48,49,50,51,52]. The roles of MEF2C and NFATc1/c2 in skeletal biology, studied in several bone cell types (osteoblast, chondrocytes, osteocytes, and osteoclasts), could in part explain the variable cellular and tissue anomalies, and phenotypes, observed in OI type V. MEF2C is a transcription factor involved in osteoblast differentiation and gene regulation, thought to be upstream of bone-specific transcription factors RUNX2 and Osterix (OSX/SP7) [53]. These two latter factors are widely accepted as master osteogenic factors that allow the differentiation of mesenchymal stem cells to osteoblasts [54]. Specifically, MEF2C binds directly to a *Runx2* enhancer leading to its osteoblast-specific expression in mice [55]. The role of MEF2C has also been explored in osteocytes, through the regulation of WNT signaling [56]. More importantly, MEF2 has also been implicated in the regulation of chondrocyte maturation and hyperthrophy [57]. The loss of MEF2C resulted in lack of hyperthophic chondrocytes and absence of endochondral bone formation, whereas MEF2C overexpression led to excessive bone formation [57]. The function of NFATc in bone is most recognized as an essential regulator of osteoclastogenesis from myeloid progenitors [58], but in osteoblasts its role is more controversial, with some opposing outcomes depending on the system used. Overexpression of a nuclear-restricted form of NFATc1 in mice leads to higher bone mass, osteoblast overgrowth and enhanced proliferation [59]. Other reports have suggested NFATc inhibits expression of osteoblast gene markers and function, and consequently a negative effect on bone formation and density [60,61,62]. It is therefore not unconceivable that exacerbated activation of MEF2 and NFATc by the MALEP BRIL in OI type V could lead to a combination of pathological consequences affecting early mesenchymal progenitors and/or differentiated osteoblast activity. Other mechanisms contributing to the effects of BRIL on NFATc activity and its inhibition by the FK506 drug could be indirectly related to the reported direct interaction of NFATc with OSX [63] and or HDAC4 [64]. These complexes were shown to bind to bone-specific genes like *Col1a1*, *Bglap*, *Ibsp*, and cooperatively modulate osteoblast differentiation.

To further explore and identify how BRIL can relay the signaling cascade to MEF2/NFATc, Y2H identified CAML as one potential intermediate highly relevant to the activation of NFATc, and possibly MEF2. Co-immunoprecipitation confirmed that both WT and MALEP BRIL interacted robustly with CAML in HEK293 and MLO-A5. CAML is a transmembrane protein of the ER that has several different functions [65,66]. It was first identified as a mediator of T-cell activation, regulating intracellular calcium levels, and activating downstream NFATc1 [67]. Despite the clear interaction of BRIL with CAML, the causality with subsequent activation of NFATc could not be firmly established as only modest effects of CAML on the Luc-reporters were measured in the MC3T3-E1, with only additive effects to those of BRIL, whereas CAML was inactive in the MLO-A5. Based on those data, it is thus not possible to ascertain a common genetic pathway (synergism) between BRIL and CAML. Yet, CAML is known to deplete intracellular Ca^2+^ stores which in turn could activate capacitance-type channels or transporters responsive to the filling state of intracellular stores [68]. The dependency of the Luc-transcriptional readouts could have been dependent on exogenous intake of Ca^2+^, which was not tested here in the presence of ionomycin or thapsigargin, to favor extracellular Ca^2+^ entry, or block re-uptake into intracellular stores. Although it is tempting to hypothesize that BRIL, through interaction with CAML, could modulate iCa^2+^ levels (or ER permeability) and therefore CnA activity, this will need to be further tested.

If confirmed, the role of CAML would also need to occur from the transient interaction of BRIL within the ER, ‘en route’ to the plasma membrane, where CAML is mainly localized [69]. Another possible scenario is that the interaction could be purely related to the tail-anchored targeting of BRIL in the secretory pathway, as we have shown previously [24]. CAML is an integral component of the ER receptor complex, mediating the integration of tail-anchored proteins like BRIL into the membrane [66]. Still, other alternative mechanisms could be envisioned, as CAML has been linked to several other cellular processes independent of its tail-anchored targeting ability [65].

One obvious mechanism caused by the MALEP BRIL, given that transcription factors are involved, would be a change in global gene expression. We have observed significant increase only caused by MALEP BRIL in the expression of targets (*Nr4a* family members, *Ptgs2*) that we previously identified as being upregulated in the bones of an OI type V knockin mouse model [35]. The fact that MALEP BRIL significantly increased activation of all 3 *Nr4a* genes could explain the corresponding increased NR4A-dependent-Luc readout. The *Nr4a (1, 2, 3)* and *Ptgs2* genes are particularly interesting given the fact that they are known to contribute to pro-inflammatory conditions [70,71]. OI type V is typified by paradoxical set of events leading to impaired primary bone formation followed by exuberant episodes of ectopic mineralization. Hyperplastic callus formation in OI type V patients can be coupled with pyrexia and tissue swelling indicating an inflammatory reaction [18,19,37,38]. Serum PGE2 is also elevated in several OI types [72]. Clinically, one of the known side effects of prostaglandin treatment for cyanotic congenital heart diseases is the appearance of cortical hyperostosis [73], a situation resembling cortical thickening and to a lesser extent the hyperplastic callus seen in OI-V and is also accompanied by increased serum alkaline phosphatase [74]. Interestingly, in a study comprising 13 OI type V individuals, those developing hyperplastic calluses had elevated plasma levels of C-reactive protein [75], indicative of systemic inflammation. PGE2 is a metabolite of the inducible COX-2, encoded by *Ptgs2*, and produced locally by osteoblasts and found important in callus formation and healing of fractures [76,77,78]. More recently, the systemic control of bone formation has convincingly been demonstrated to occur in part by expression of COX-2 and formation of PGE2 by osteoblasts through signaling to sensory nerves which in turn control sympathetic tone and bone formation [78,79]. The transcription of *Ptgs2* has been studied extensively and is responsive at least to NFATc1 activity [80,81]. On the other hand, MEF2s and NFATcs, have been shown to activate *Nr4a* genes [82,83]. Whether the increased *Ptgs2* and *Nr4a* are directly upregulated by the activated MEF2 and/or NFATc activity warrants further investigation. Additionally, the existence of a potential interplay between the COX-2 (PGE2) and accentuated NR4A activity is possible. It has recently been demonstrated that PGE2 can be a ligand for NR4A orphan receptor family members, promoting their DNA binding ability and transcriptional activity [84]. Future studies including chromatin immunoprecipitation coupled to global RNAseq will be required to formally validate binding of the MEF2, NFATc, and possibly NR4A factors to regulatory elements and identify the whole spectrum of genes activated by MALEP BRIL.

The reasons why the MALEP BRIL would be more conducive at stimulating the pathways remain to be established. Because WT and MALEP BRIL, when expressed to similar levels, are mainly localized at the plasma membrane, in addition to ER-Golgi, their N-terminal intracellular moiety in close proximity with the plasma membrane is likely to be the source of the effect. The MALEP BRIL extension could serve as a better docking site for the interaction with CAML, or another unidentified interactor, but the kinetics and/or dynamics of such interactions will require more quantitative approaches. The therapeutic applicability of our findings in OI type V may be envisioned next. In that respect, Hanagata et al. [36] have investigated whether in vivo treatment with FK506 in a model of OI type V could inhibit *Ifitm5* expression, and/or its interaction with FKBP11 that they had previously identified [25]. They showed that although FK506 administration in embryos improved bone mineral content in the mutant bones, it neither prevented the peri-natal lethality nor rescued the severe skeletal malformations [36]. These results may point to the inefficacy of FK506 in this specific context of a curative approach in the OI type V mouse models [34,35,36] that are strikingly more severe phenotypically than the patients. The timing of treatment might also need to be re-visited in a prophylactic approach as BRIL is expressed in mice as early as E13.5, and possibly generating already profound effects by the time FK506 effects manifest.

Irrespective of the possible different mechanisms operating, the data presented here are the very first ever to be reported for an ‘activity’ or a ‘readout’ for BRIL. Our results also support the idea that the pathogenic MALEP BRIL is due to a gain-of-function. Although further investigation into the relationship between WT and MALEP BRIL and downstream intracellular factors is needed, the concepts raised herein provide a solid framework to test for the pathologic consequences of MALEP BRIL in OI, and possibly to investigate some of the other mutations reported in BRIL. As for the potential clinical implications, it is currently unclear if drugs inhibiting the identified pathways, such as FK506 or anti-inflammatory molecules, could be useful prophylactically to prevent, or to alleviate, some of the clinical manifestations. Further testing on relevant animal models may help explore these different possibilities.

## 4. Materials and Methods

### 4.1. Cell Culture and Transfection

All cell lines (MC3T3-E1 mouse pre-osteoblast subclone 14; HEK293 embryonic kidney; MLO-A5 mouse pre-osteocytic; SaOS2 human osteosarcoma; UMR106 rat osteosarcoma) were used within a maximum passage number of 20. The MC3T3-E1 were grown in alpha-MEM (Thermo Fisher, Waltham, MA, USA; 12561056) and all others in DMEM (Life Technologies, Carlsbad, CA, USA; 11965092) supplemented with 10% FBS (Thermo Fisher, Waltham, MA, USA, Gibco Qualified). Media were changed every 2–3 days. Depending on the downstream application, cells were seeded in 24-well (luciferase assay) or 6-well plates (RNA/protein extraction/co-IP) format (Sarstedt, Sarstedt, Germany; 83.3920 and 83.3922) at 22,000 cells/cm^2^ (MC3T3-E1 and MLO-A5) or 33,000 cells/cm^2^ (HEK293, SaOS-2, UMR106). Cells were approximately 70% confluent at the time of transient transfection, carried out 24 h post-seeding. Transfections were done with Xtreme Gene 9 Transfection Reagent following the instructions recommended by the manufacturer (Millipore Sigma Oakville, Oakville, ON, Canada) using a ratio of 6 μL per 1 μg of DNA for MC3T3-E1 cells and 3 μL per 1 μg of DNA for all others. The total amount of plasmid DNA transfected per well was always kept constant (0.2 μg to 0.3 μg/24-wells; 1 μg/6-wells) and completed with a GFP-expressing plasmid when appropriate for dual- or triple-transfections. The GFP plasmid was also used as a negative control for normalization of luciferase activity or gene expression monitoring.

### 4.2. Cloning and Luciferase (Luc) Reporter- and Expression-Plasmids

The characteristics and source of all Luc reporters, expression plasmids, and oligonucleotides sequences are presented in the Supplemental Tables S1–S3. All expression plasmids have the pcDNA3.1 backbone and have been validated by Sanger sequencing using the services of Centre d’expertise et de services Génome Québec (https://cesgq.com/home, accessed on 10 January 2022).

### 4.3. Luc Assay

Luciferase assays were carried out 24 h post-transfection unless otherwise indicated. The cells were washed once with PBS and lysed using 0.1 mL of passive cell lysis buffer (Promega, Madison, MI, USA; E1941). After 10 min incubation on a rocker plate, 5 µL sample of the lysed cells was mixed with 50 µL of Luciferase Assay Reagent (Promega; E1501) dispensed in 5 mL polystyrene luminometer tubes (Sarstedt; 55.476.305). Light output was measured using a Sirius luminometer (Berthold, Oak Ridge, TN, USA) programmed to perform a 2-s measurement delay followed by a 10-s measurement read for luciferase activity. Each transfection was done in triplicate wells, with 3–6 individual biological replicates.

### 4.4. RNA Extraction and RT-qPCR

RNA extractions were carried out 24 h post-transfection using the Trizol reagent (Millipore-Sigma) following the manufacturer recommendations. Briefly, cells were washed 3 times with PBS. Trizol was added (1 mL/well) and the cell lysates were pipetted up-and-down several times and transferred in a microtube. Cell lysates were homogenized by vortexing for approximately 1 min. Chloroform (0.2 mL) was added to the homogenates, vortexed again for 1 min and incubated at room temperature for 5 min. The samples were then centrifuged for 15 min at 13,200 rpm at 4 °C. The upper aqueous phase was recovered, the RNA was precipitated with 1 volume of isopropanol and centrifuged at 13,200 rpm for 10 min at 4 °C. RNA pellets were washed using ethanol 70%, briefly dried and resuspended in RNAse-free water. The purity and concentration of the RNA samples was assessed on a NanoDrop 1000 Spectrophotometer (Thermo Fisher, Waltham, MA, USA) before storing at −20 °C until use.

Complementary DNA (cDNA) was prepared from the extracted RNA samples using the High Capacity cDNA Reverse Transcription Kit (Thermo Fisher, Waltham, MA, USA). Reactions of 20 μL containing 2 μg of RNA were prepared according to the manufacturer’s protocol using the following conditions: 25 °C for 10 min, 37 °C for 2 h and 85 °C for 5 min. Resulting cDNA was diluted 1/5 with Milli-Q water and used for real-time PCR in reactions of 20 μL in duplicates prepared in MicroAmp Optical 96-well Reaction Plates (Thermo Fisher, Waltham, MA, USA) using TaqMan Fast Advanced Master Mix and TaqMan Gene Expression Assay Probes (Applied Biosystems) found in Supplemental Table S4. Plates were run and analyzed on a 7500 Real Time PCR System (Applied Biosystems) using the Relative Quantification (ΔCt) plate assay function of the 7500 Sequence Detection Software v.1.3.0. Taqman probe for mouse β-actin (*Actβ*) was used as endogenous gene control.

### 4.5. Protein Extraction, Western Blotting and Co-Immunoprecipitation

Protein extractions were carried out 24 h post-transfection. Cells were washed 3 times with PBS at RT, and collected with a cell scraper using 100 μL/6-well of 50 mM Tris-HCl (pH 7.4), 150 mM NaCl, 1 mM EDTA, 1% (*v*/*v*) NP-40 (nonylphenoxypolyethoxyl-ethanol) with 1× of protease inhibitor cocktail (Sigma Aldrich, Oakville, ON, Canada). Cell lysates were transferred to a 1.5 mL microtube, incubated on ice for 10 min and vortexed at 2-min intervals. Homogenates were cleared by centrifugation at 13,200 rpm for 10 min at 4 °C. Soluble proteins from the homogenate supernatant were recovered. For direct Western blotting, samples were combined with 4× Laemmli buffer (200 mM Tris-HCl pH 6.8, 8% SDS, 40% glycerol, 50 mM EDTA, 0.08% bromophenol blue) with 5% β-mercaptoethanol, boiled for 5 min and stored at −20 °C until analysis. For anti-BRIL immunoblotting, equal protein sample volumes were loaded on home-made 1 mm thick 15% SDS-PAGE (BioRad, Mississauga, ON, Canada; Miniprotean) and migrated in Tris-glycine SDS running buffer. Proteins were transferred to 0.45 μm nitrocellulose membranes (Sigma Aldrich, Oakville, ON, Canada, Amersham Protran) and efficiency of transfer monitored by Ponceau S staining. Membranes were blocked in PBS with 5% skim milk and 0.05% Tween for 1 h and incubated overnight with the indicated primary antibody at 4 °C. Membranes were washed thrice for 10 min with PBS-Tween 0.05% and treated with the secondary HRP-coupled antibodies (diluted 1/30,000 in blocking solution) for 1 h at room temperature. Detection was done using ECL Prime Western Blotting Detection Reagent (GE Healthcare). Both primary and secondary antibodies were diluted in the blocking solution. The rabbit-anti-BRIL antibody, which detects an N-terminal epitope (DTSYPREDPRAPSS) as described previously [16], was used at a 1/4000 dilution (4 μg/mL). Hybridization with an alpha-tubulin (DSHB; clone 12G10) monoclonal antibody was used to normalize for total protein content. The donkey anti-rabbit-HPR-coupled (GE Healthcare; NA934V) and a sheep anti-mouse-HRP-coupled (Sigma; A6782) secondary antibodies were used at 1/30,000.

For the co-immunoprecipitation experiments, all steps and incubations were performed at 4 °C. Cell extracts were prepared as described above, and for the total cell lysate, a 10 μL aliquot was mixed with 10 μL of 2× Laemmli sample buffer, boiled for 2 min, and frozen at −20 °C until use. The remaining 90 μL was incubated with 8 μg of rabbit-anti-BRIL or rabbit-anti-FLAG (Millipore-Sigma, Oakville, ON, Canada; F7425) antibodies for 2 h. Then 35 μL of pre-equilibrated dynabeads-protein-G slurry (Thermo Fisher, Waltham, MA, USA): 10004D was added and incubated for 1.5 h. The captured bead-antibody-protein complex was recovered with a Dyna-Mag2 magnet (Thermo Fisher) and washed 3 times with 1 mL each of cell lysis buffer. The immunoprecipitated proteins were eluted by incubation with 70 μL of acidic buffer (glycine 0.1 M pH 2.0) for 5 min at RT° with occasional mixing. The eluted material was recovered, neutralized with 3 μL of Tris-HCl 1 M (pH 7.5), and mixed with 25 μL of 4× Laemmli sample buffer before being boiled for 5 min. Proteins were separated on NuPAGE 4–12% gradient gels (Thermo Fisher; NP0336) using 1x MOPS-SDS running buffer (Thermo Fisher; NP0001). Proteins were transferred to Protran 0.45 μm nitrocellulose for 1 h at 40 V at RT with transfer buffer (Thermo Fisher; NP0006). Sequential immunoblotting (anti-FLAG; anti-BRIL) was done as described above, and after stripping for 20 min at RT° with a buffer containing 200 mM glycine (pH 2.2), 0.1% (*w*/*v*) SDS, 0.1% (*v*/*v*) NP40.

### 4.6. Yeast 2-Hybrid (Y2H) Screen

The human WT and MALEP BRIL coding sequences covering the cytoplasmic region only (a.a. 1-86 and 1-91, respectively) were sub-cloned in frame at the N-terminus of GAL4-AD in the pGBK (Takara Bio, San Jose, CA, USA) backbone plasmid for use as baits. The identity of the baits was additionally confirmed by colony PCR and sequencing after yeast transformation. All screens were performed by Next Interactions Inc. (Richmond, CA, USA) with a mating-based Y2H approach and in 2 series of triplicates (total 6 repeats). A corresponding series of control screens was undertaken using the empty vector, or with 2 unrelated baits. Selection for all screens was on low stringency medium SD-LTH (HIS3 activation only). At the end of the selection process, prey cDNA inserts were prepared for Next-Generation Sequencing (NGS). Sample prep was done with a standard procedure of low-cycle amplification of prey inserts and modified Nextera method from Illumina. The readout was done on lanes of Illumina HiSeq2000 using 50SR single read with indexing. Total sample number was 21 consisting of 6 repeat screens for WT-BRIL, MALEP BRIL and controls. The screen for prey was performed using the cDNA library Matchmaker Human Normalized Universal (Takara Bio, San Jose, CA, USA; 638874). Analysis of mapped reads per gene (see Supplemental Table S5) revealed that ~13,000 of the 23,393 *H. sapiens* genes in ENSEMBL were represented with 1 or more reads, and ~10,000 by 10 or more reads. There was absence of self-activation of the baits (<1 per million bait expressing cells), as measured solely for HIS3 activation. For the NGS analysis, a high stringency cutoff pipeline was applied with log2FC >1 (effective 2-fold change) as primary, and adjusted *p* value < 0.05 (95% probability) as a secondary criterion for all comparisons of WT and MALEP BRIL with background controls. The identity of the high confidence interaction quantitation/analysis method was designed to maximize the chance to discover any potential positive genes. This was done by binning the positive genes that have bait enrichments >25% above the pool average control values into one of three tiers for WT and MALEP, depending on 3 parameters: fold change, p value, or the adjusted pooled average. The three tiers had the following order of hit confidence: 1 > 2 > 3. All raw data and comparisons are given in the Supplemental Table S5.

### 4.7. Statistical Analysis

Data are expressed as mean and standard deviation (SD) or standard error of the mean (SEM), as indicated. Each experiment included three repeats (independent wells), and biological replicates (independent experiments) ranged from 3 to 6, depending on the experiment. Statistical significance was assessed using, as appropriate, one-way or two-way ANOVA, depending on the data. This was followed by Tukey’s multiple comparisons test. For direct comparisons between 2 samples, an unpaired one-tailed t-test was performed with a statistical p value set at ≤0.05. Statistical analyses were performed utilizing GraphPad Prism 6 (GraphPad Software).

## Data Availability

All data are within the present article and Supplementary Material.

## Supplementary Materials

The following supporting information can be downloaded at: https://www.mdpi.com/1422-0067/23/4/2148/s1.
