# Supplementary material for: The Osteogenesis Imperfecta Type V Mutant BRIL/IFITM5 Promotes Transcriptional Activation of MEF2, NFATc, and NR4A in Osteoblasts
Source: Int J Mol Sci. 2022 Feb 15;23(4):2148. doi: 10.3390/ijms23042148 (PMC8875491; doi:10.3390/ijms23042148)
Supplement: Supplementary file 1 [file ijms-23-02148-s001.zip › Non-cropped images IJMS.pdf]

Pertaining to Figure 2E

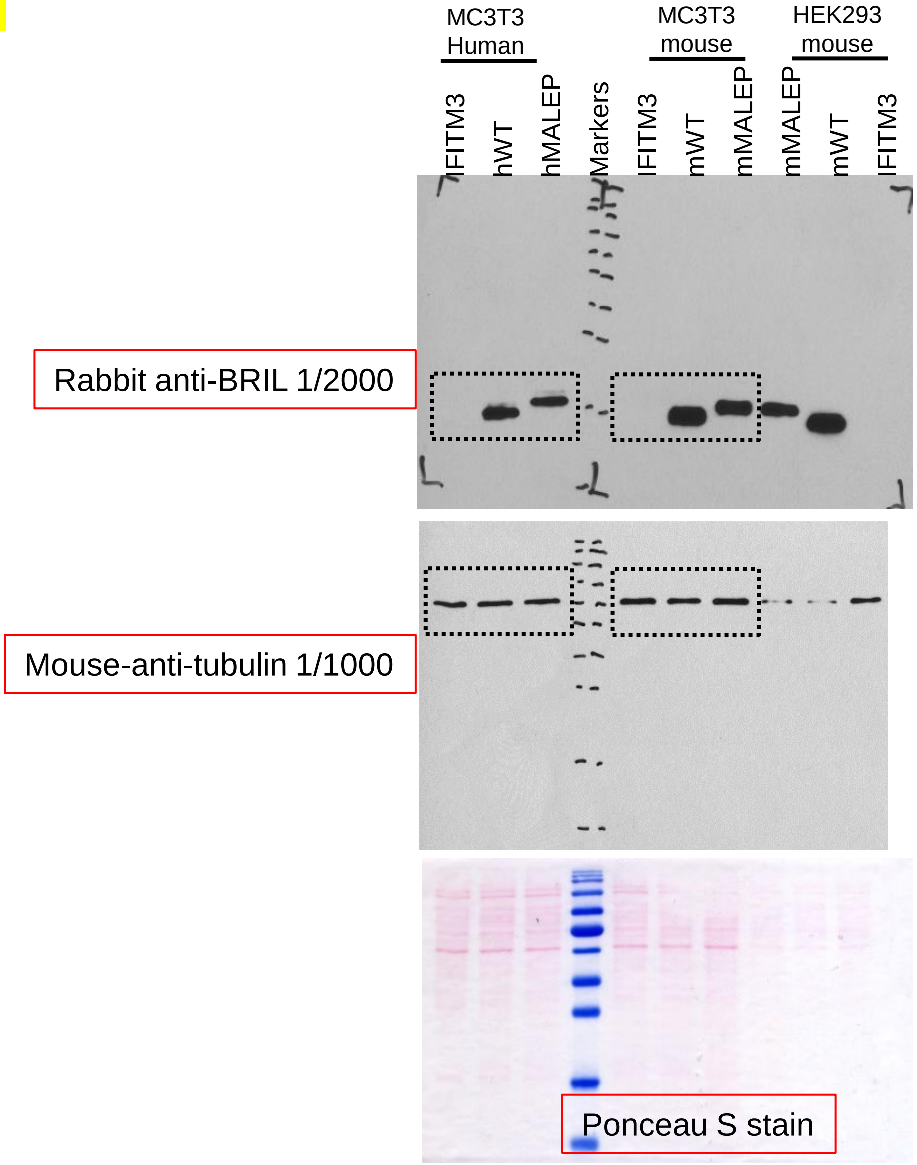

Pertaining to Figure 3E

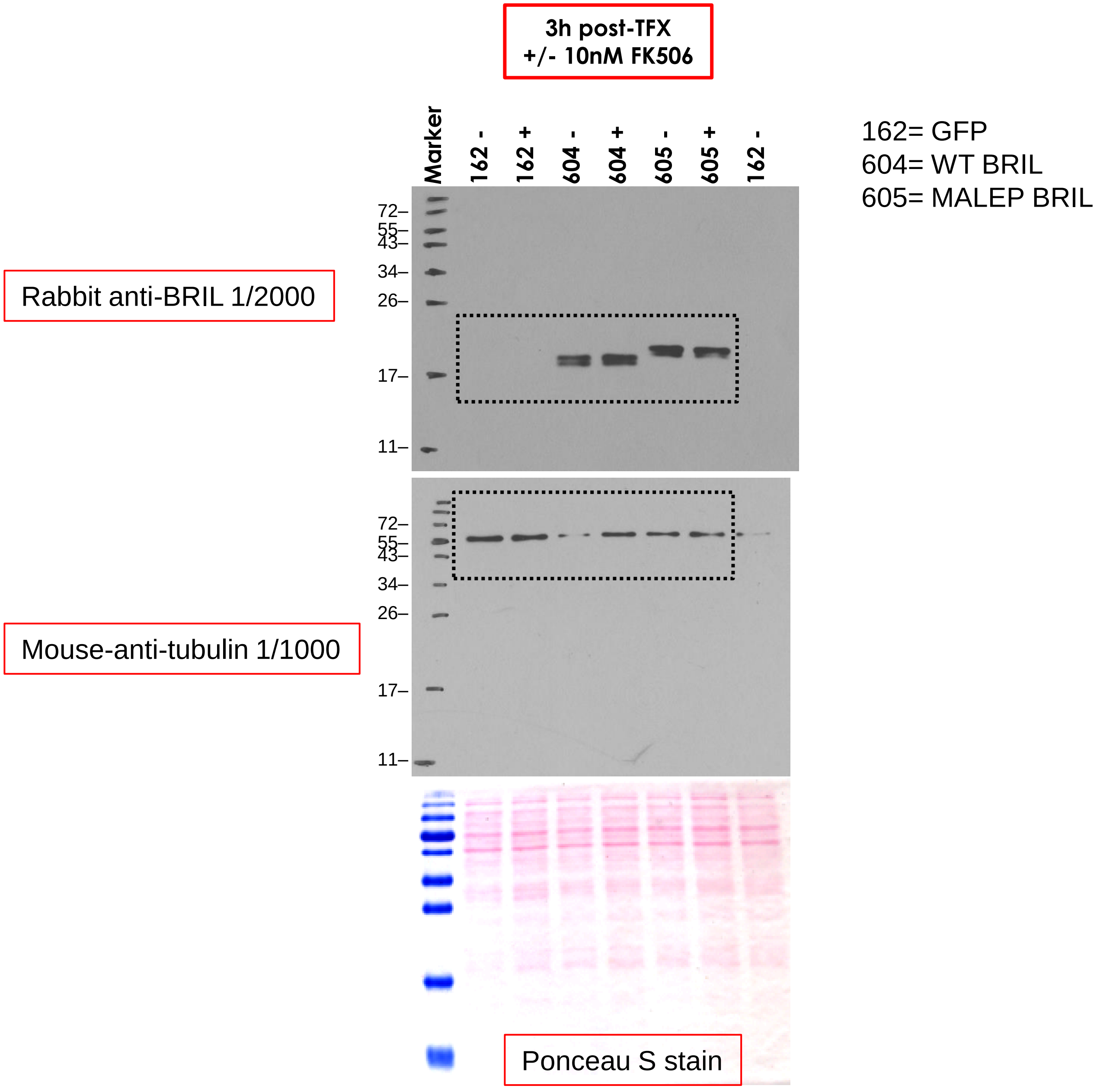

Pertaining to Figure 5C

162= GFP
604= WT BRIL
605= MALEP BRIL

Rabbit anti-BRIL 1/2000

Mouse-anti-tubulin 1/1000

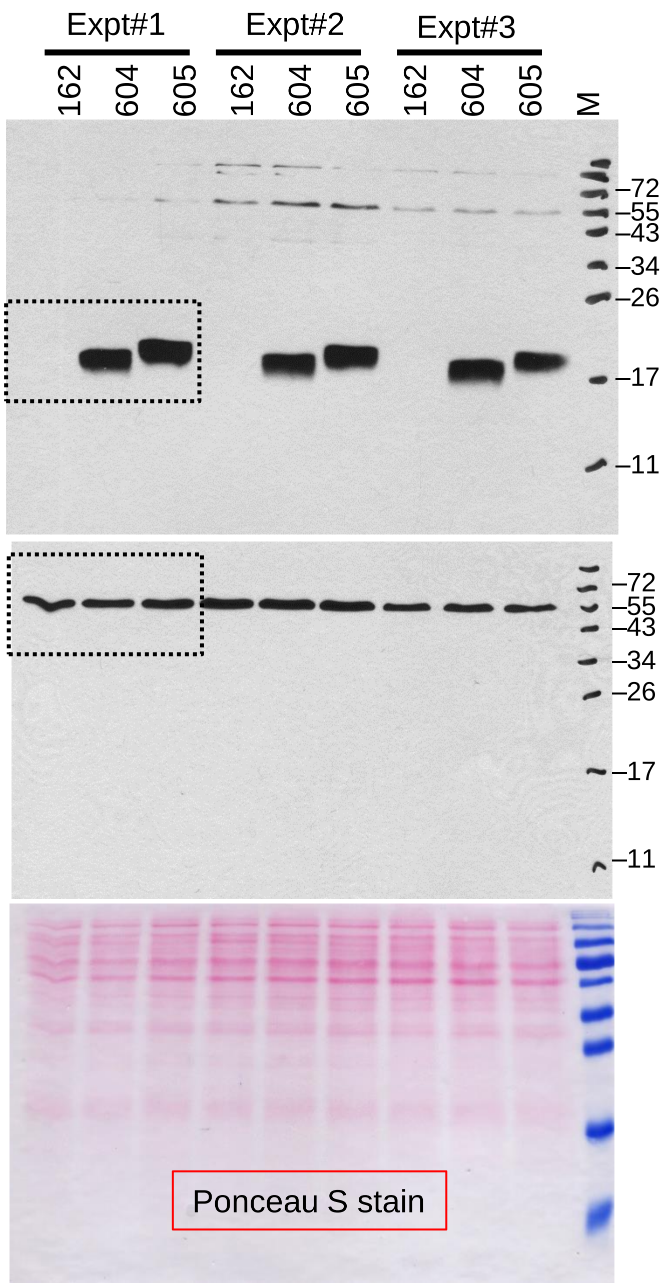

Pertaining to Figure 5D

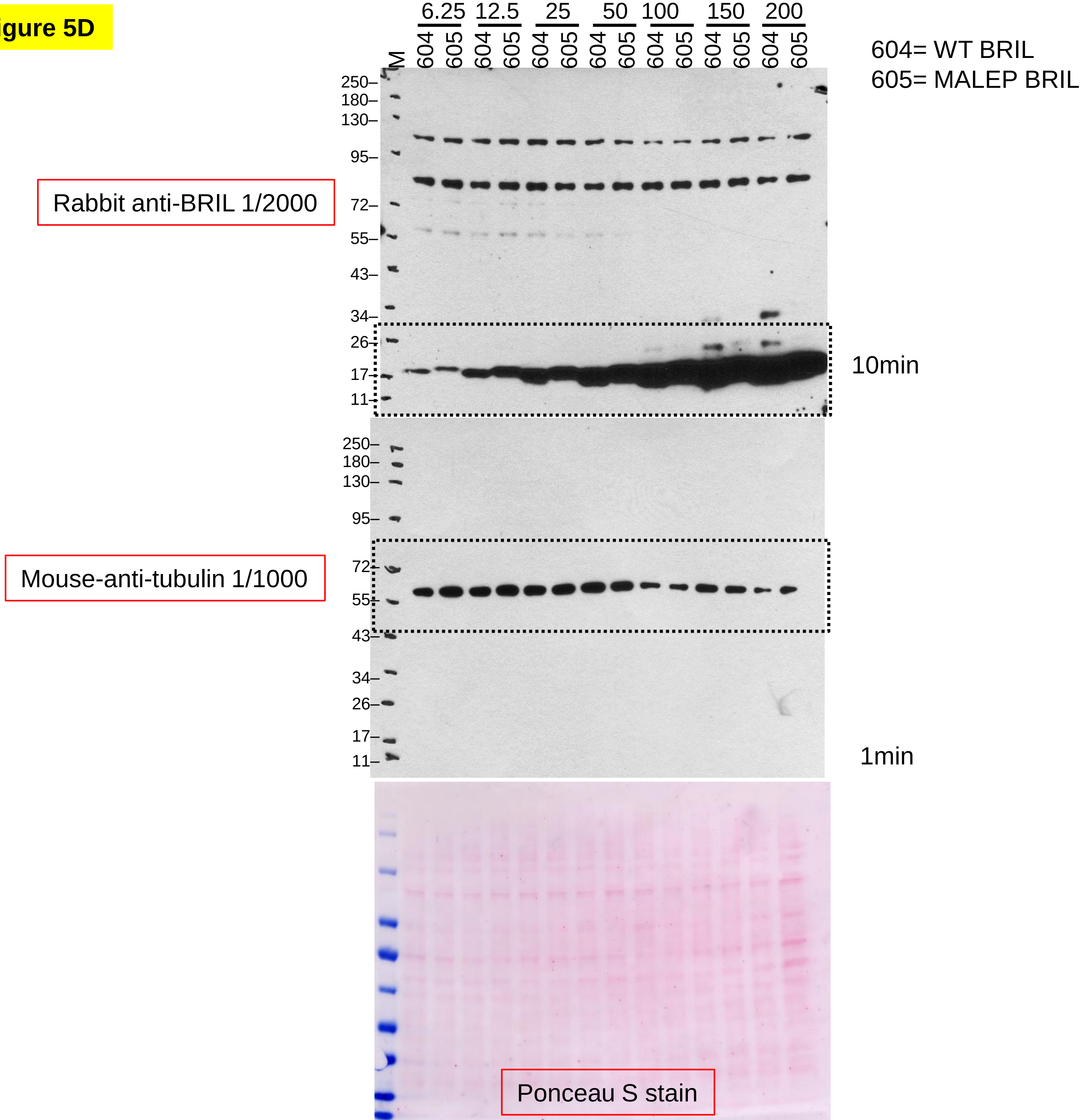

Pertaining to Figure 6C

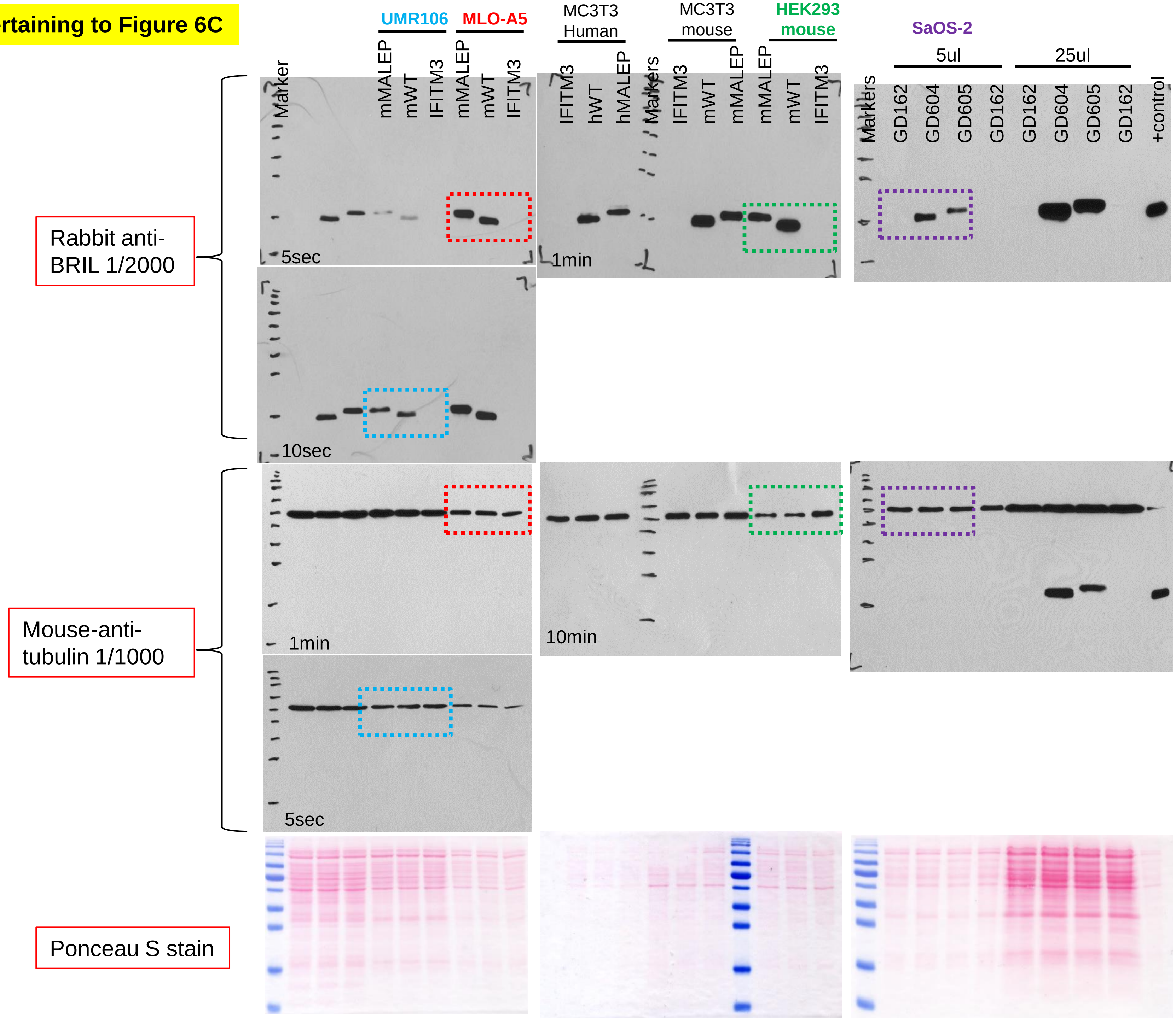

Pertaining to figure 9A (HEK293)

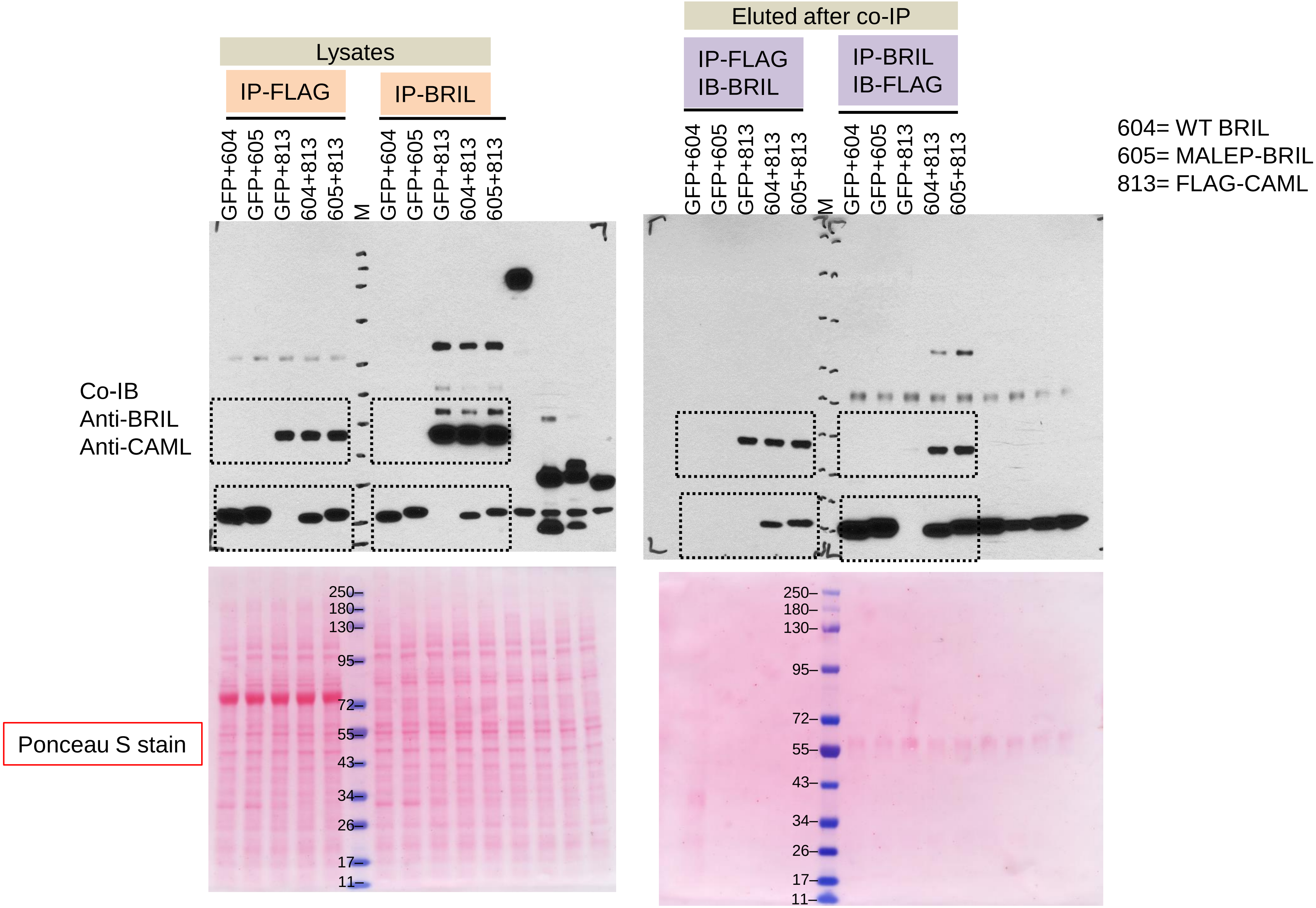

Pertaining to figure 9B (MLO-A5)

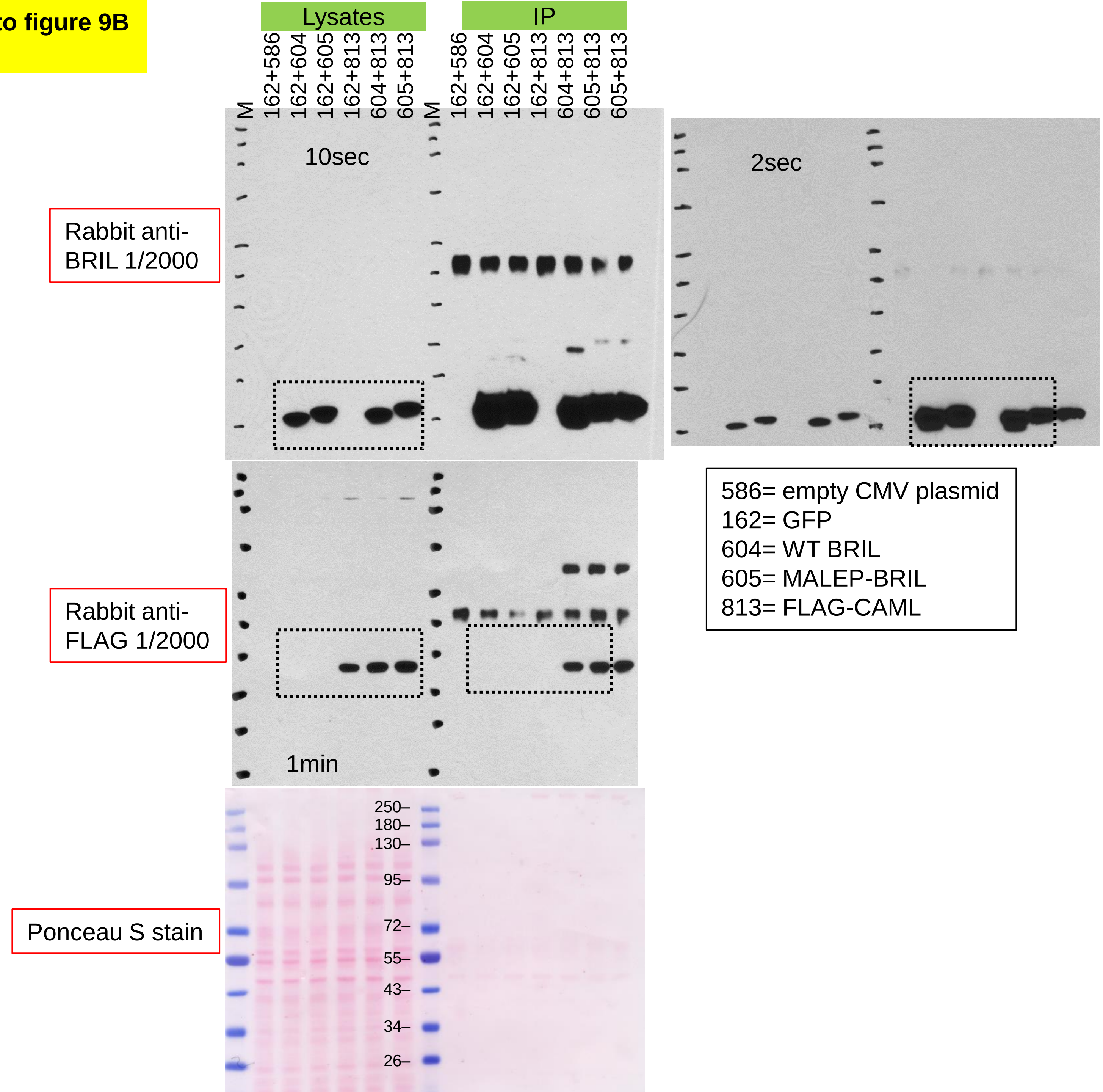

586= empty CMV plasmid
162= GFP
604= WT BRIL
605= MALEP-BRIL
813= FLAG-CAML
