# Supplementary material for: The Osteogenesis Imperfecta Type V Mutant BRIL/IFITM5 Promotes Transcriptional Activation of MEF2, NFATc, and NR4A in Osteoblasts
Source: Int J Mol Sci. 2022 Feb 15;23(4):2148. doi: 10.3390/ijms23042148 (PMC8875491; doi:10.3390/ijms23042148)
Supplement: Supplementary file 1 [file ijms-23-02148-s001.zip › Supplemental Figures S1 and S2.pdf]

The osteogenesis imperfecta type V mutant BRIL/IFITM5 promotes transcriptional activation of MEF2, NFATc, and NR4A in osteoblasts.

Vincent Maranda [1], Marie-Hélène Gaumond [1] and Pierre Moffatt [1,2,3,*]

Supplemental Figures S1 and S2

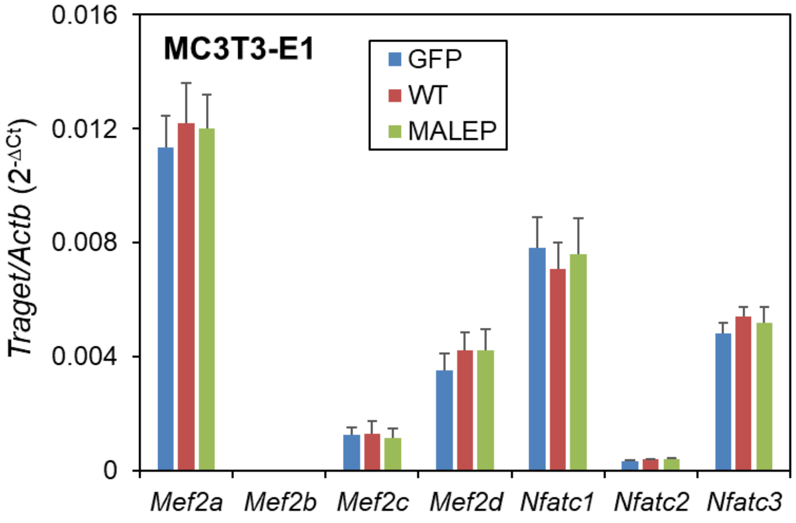

Figure S1. Gene expression monitoring for *Mef2* and *Nfatc* by qPCR in transfected MC3T3-E1 cells. Total RNA was collected 24 h after transfection with expression plasmids encoding GFP, WT- and MALEP BRIL in MC3T3-E1. RT-qPCR was performed to analyze the various targets indicated. Expression levels are expressed as $2^{-\Delta Ct}$ as normalized to β-actin (*Actβ*). Data represents average ± SD (n=4).

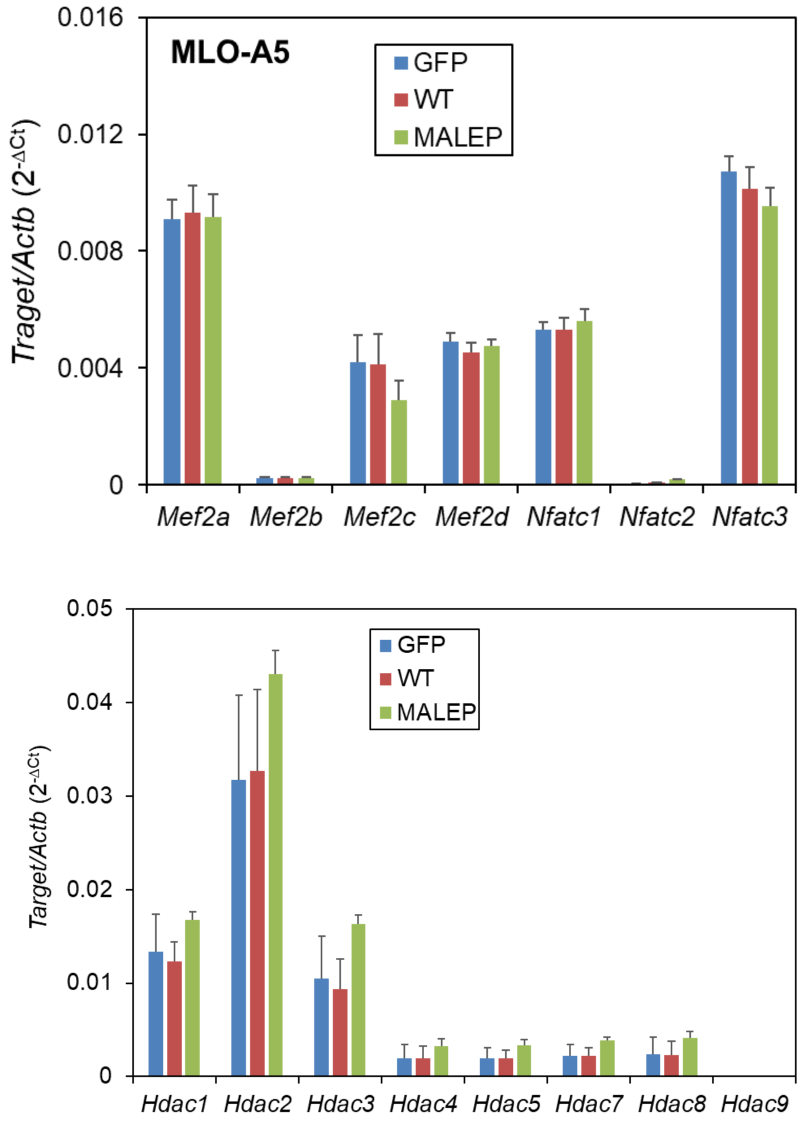

Figure S2. Gene expression monitoring for *Mef2*, *Nfatc, and Hdac* by qPCR in transfected MLO-A5 cells. Total RNA was collected 24 h after transfection with expression plasmids encoding GFP, WT- and MALEP BRIL in the MLO-A5 cells. RT-qPCR was performed to analyze the various targets indicated (top panel, *Mef2* and *Nfatc*; bottom panel, *Hdac*). Expression levels are expressed as $2^{-\Delta Ct}$ as normalized to β-actin (*Actβ*). Data represents average ± SD (n=3).
